# mHealth Apps Available in Italy to Support Health Care Professionals in Antimicrobial Stewardship Implementation: Systematic Search in App Stores and Content Analysis

**DOI:** 10.2196/51122

**Published:** 2025-04-29

**Authors:** Giuseppa Russo, Annachiara Petrazzuolo, Marino Trivisani, Giuseppe Virone, Elena Mazzolini, Davide Pecori, Assunta Sartor, Sergio Giuseppe Intini, Stefano Celotto, Rossana Roncato, Roberto Cocconi, Luca Arnoldo, Laura Brunelli

**Affiliations:** 1 Department of Medicine, University of Udine, Via Colugna 50, Udine, 33100, Italy, 39 3492131278; 2 Department of Epidemiology, Experimental Zooprophylactic Institute of Venezia, Udine and Legnaro, Legnaro, Italy; 3 Infectious Disease Unit, Friuli Centrale Healthcare University Trust, Udine, Italy; 4 Microbiology Unit, Friuli Centrale Healthcare University Trust, Udine, Italy; 5 General Surgery Clinic and Liver Transplant Center, Friuli Centrale Healthcare University Trust, Udine, Italy; 6 Primary Care Department, Friuli Centrale Healthcare University Trust, Udine, Italy; 7 Accreditation, Quality, and Clinical Risk Unit, Friuli Centrale Healthcare University Trust, Udine, Italy

**Keywords:** antimicrobial resistance, antimicrobial stewardship, mHealth, app, prescribing behaviors, one health, antimicrobial, Italy, application, support, therapy support, diagnosis, engagement, mobile health

## Abstract

**Background:**

Antimicrobial resistance (AMR) is a major challenge of the 21st century. Digital technologies are now an increasingly effective means of supporting optimal health care delivery and public health.

**Objective:**

The aim of this study was to explore the apps available to support health care professionals in the fight against AMR.

**Methods:**

A total of 4 independent researchers conducted a systematic search of the App Store and Google Play Store using the following keywords: “antimicrobial resistance,” “antibiotic resistance,” “antimicrobial stewardship,” “antibiotic stewardship,” “antibiotic guide,” “antibiotic therapy,” and “antimicrobial therapy.” The same keywords were also searched in Italian. The apps whose contents were in languages other than Italian or English, or apps which were games, or had multimedia or paid content and advertising, or apps for only specific pathologies were not considered. A set of basic information was collected for all apps found. After downloading the apps, they were evaluated using an 86-item checklist containing expert-validated criteria aggregated in the domains of pathogens and etiological agents, diagnosis and therapy support, AMR, dashboard function, antimicrobial stewardship (AMS), notes and recordings, network, and technical characteristics of the app.

**Results:**

First, 115 apps were identified: 31 apps for Android and 84 apps for iOS. By applying the exclusion criteria, 31 apps were excluded (16 for Android and 15 for iOS) for the following reasons: not available in Italian or English (6 apps), not freely available (14 apps), required registration (5 apps), and games (6 apps). The remaining 84 eligible apps (15 for Android and 69 for iOS) were downloaded, installed, and further analyzed using the same criteria, excluding 57 apps (48 for iOS and 9 for Android) for the following reasons: required further registration (16 apps), language other than Italian or English (17 apps), pathology specific (5 apps), paid content (8 apps), specific to veterinarians (4 apps), recreational apps (2 apps), referred to only scientific articles (1 app), no longer available (1 app), and not health care objectives (3 apps). The remaining 27 apps (6 for Android and 21 for iOS) were selected for in-depth analysis. Of the 27 apps that met the inclusion criteria, most apps did not fulfill the desirable aspects and only 2 of them achieved a fulfillment score of 36%. The highest scores were achieved for support for diagnosis and therapy (37%) and technical characteristics of the app (23%). Lower scores were achieved for AMS (8%), pathogens and etiological agents (4%), notes and records (3%), network (2%), AMR (1%), and dashboard function (1%).

**Conclusions:**

None of the apps examined successfully provided the desired features and functions. To better engage of prescribers in the fight against AMR, the development of an app that meets the requirements is needed.

## Introduction

Antimicrobial resistance (AMR) is a serious problem [1] and remains one of the greatest global threats to public health at the beginning of the 21st century [2]. There is no single solution that can solve the problem alone, but a so-called theragnostic model based on infection prevention, antibiotic stewardship, and diagnostic stewardship programs is required [3]. It is estimated that bacterial infections will cause around 10 million deaths per year worldwide in 2050, far exceeding deaths from cancer (8.2 million), diabetes (1.5 million), or traffic accidents (1.2 million), with projected costs exceeding US $100 trillion [4]. In 2019, up to 4.95 million people died from diseases in which bacterial AMR played a role. Of these, 1.27 million deaths were the direct result of AMR—meaning that drug-resistant infections killed more people than HIV/AIDS (864,000 deaths) or malaria (643,000 deaths) [5]. In the United States alone, more than 2 million people become infected with multidrug-resistant organisms each year, resulting in 23,000 deaths per year, and the fact that the cost of antimicrobial resistance in the United States is estimated at US $20 billion per year underscores the need for action [6,7]. In addition, in the European Union (EU) and in the European Economic Area (EEA), there are more than 670,000 infections caused by antibiotic-resistant bacteria every year, and about 33,000 people die, as a direct result of these infections [8]. In Italy, antibiotic resistance remains one of the highest in Europe, and in our country up to 10% of patients contract multidrug-resistant bacterial infections every day, resulting in thousands of deaths. In Italy, about 284,100 patients are affected by health care–associated infections every year, resulting in about 4500‐7000 deaths [9].

It is known that the inappropriate use of antimicrobials leads to unnecessary exposure of pathogens to antimicrobials, which over time leads to the development of AMR [10]. It is estimated that a mixed package of measures including responsible antibiotic use programs, improved hygiene, mass media campaigns, and the use of rapid diagnostic tests could prevent around 27,000 deaths per year in the EU and EEA [11]. However, the problem of antimicrobial resistance does not only affect the human sector, but also the environmental and veterinary sectors, where antibiotics are also widely used, and the impact of antimicrobial resistance is equally significant. Therefore, combating AMR cannot be done independently of a “One Health” approach that promotes coordinated action in the different sectors concerned [12]. As the World Health Organization (WHO) points out, the problem of AMR could also have a significant impact on a number of Sustainable Development Goals (SDGs). For example, the 2030 Agenda includes two specific indicators for AMR under SDG 3 (good health and well-being), including number 3.2—percentage of bloodstream infections due to selected antimicrobial-resistant organisms and number 3.3—proportion of health facilities that have a core stock of relevant essential medicines that are sustainably available and affordable [13].

In addressing such an impressive morbidity and mortality burden, digital mobile health (mHealth) technologies could play a crucial role in this worrying scenario. In fact, AMR counseling and support could be effectively aided by the use of information technology, big data, and artificial intelligence (AI) in health care. Despite debates over terminology, mHealth encompasses various types of solutions that could support medical and public health practices using mobile devices, such as cell phones, patient monitoring devices, personal digital assistants, and other wireless devices [14]. Such digital technologies are becoming an important resource for healthcare service delivery and public health. Among them, mobile wireless technologies are particularly important due to their ease of use, wide reach, and broad adoption [15]. To support health care professionals in antimicrobial stewardship, workflow integration with systems that use electronic health records (EHRs), including specific software or predictive AI, could be a good solution, but in areas where technology is not as advanced in the use of HHR or where access to EHR is limited or unavailable, the use of mobile phones, especially well-developed apps that support health care workers in antimicrobial stewardship (AMS), could be an effective key. Today, a large proportion of innovative digital health solutions come from start-ups that are building their services and technologies on Apple and Google mobile app platforms, using powerful analytics based on AI and machine learning, and developing new patient-oriented business models [16]. Among these solutions, smartphone apps are among the top 10 digital health technologies expected to impact the workforce between 2020 and 2040 [17]. Given this complex and challenging scenario, the aim of our study was to evaluate the mHealth apps available in Italy to support health care professionals and in particular prescribers, in the fight against AMR.

## Methods

### Step 1: Systematic Search

A systematic search was conducted by 4 independent researchers from the University of Udine, Italy, for the selection, data extraction, and functional evaluation of antimicrobial resistance apps available on the Apple App Store and Google Play Store. The Italian and English terms for antimicrobial resistance, antibiotic resistance, antimicrobial stewardship, antibiotic stewardship, antibiotic guide, antibiotic therapy and antimicrobial therapy were used as keywords in both stores. All apps that were found with at least 1 keyword mentioned above were included in the study. The choice to start the systematic search using keywords was necessary because there are too many healthcare apps and it was essential to reduce the action area. The following exclusion criteria applied to the search: content in languages other than Italian or English, games, leisure apps, photo and video apps, paid apps, paid content, advertising, apps that require registration, apps that only contain references to scientific articles, and apps that are not focused on health care. Paid apps and paid content were excluded as the focus of the research was to find apps that support health care professionals in the implementation of AMS and are, therefore, accessible to all health care professionals regardless of their financial means or affiliation with a public or private institution. Pathology specific apps were excluded as the aim of the study was to find a generic app that covers all diseases and systems. A set of basic information was collected for all apps found. This included the name of the app, operating system (iOS or Android), availability of additional paid content, size (MB), number of downloads, position in the download ranking, user rating, copyright, privacy policy, possible presence of European medical device (CE) labelling, number of languages, store category, recipients, date of first release, last update, and responsibility of support. A total of 4 researchers conducted the search independently between June 15, 2022, and October 18, 2022, (2 researchers worked with iOS and 2 researchers worked with Android). All the contradictory information collected was discussed until a consensus was reached. Then the results were merged and app deduplication was performed.

### Step 2: App Evaluation

A first draft of a checklist was created, listing the app features that were considered ideal for the app users. The main topics (domains) to be evaluated were related to the content of the app, notes and records, social support, and technical features. Individual questions were formulated for each domain based on the scientific literature [2,7,12] and the authors’ previous experience in the field of mHealth apps for postnatal care [18] and for antenatal and postnatal care [19]. Possible answers were yes (y), no (n), or partially (p). The first draft of the checklist was commented on by a group of experts involved in AMR at different levels, including infectious disease specialists, microbiologists, clinicians, public health and infection risk management specialists, pharmacologists, and veterinarians. The valuable multidisciplinary collaboration between these professionals [20] helped to refine the assessment tool for the final draft of the 86-item checklist, which covers 8 domains, namely: pathogens and etiological agents, diagnostic and therapeutic support, AMR, dashboard function, AMS, notes and records, network, and technical features. Table 1 shows the final version of the checklist.

**Table 1.** Checklist for apps to support antimicrobial stewardship (AMS).

Domain	Item
Pathogens or etiological agents	1. Does the app contain information about the main microbiological characteristics of microorganisms?2. Does the app contain information about the characteristics of aerobic and anaerobic microorganisms?3. Does the app contain information about the cell wall of microorganisms?4. Does the app contain information about the main characteristics of fungal pathogens?5. Does the app contain information about the availability of vaccines that can prevent infections/diseases caused by infectious agents?6. Does the app include information about the methods and timeline for reporting infectious diseases?7. Does the app provide information on how antimicrobial drugs work?8. Does the app provide guidance on infection prevention and control (personal protective equipments, procedures)?
Diagnostic and therapeutic support	9. Does the app contain information about infections and their etiology?10. Does the app include information to help distinguish between a community-acquired infection (CAI) and a health care–acquired infection (HAI)?11. Does the app include references to diagnostic tests that are useful in formulating the diagnosis?12. Does the app support the user in selecting a drug for empiric therapy according to the clinical condition?13. Does the app indicate the route of administration of the medications indicated for therapy?14. Does the app provide specific information on drug pharmacokinetics and pharmacodynamics?15. Does the app indicate the expected duration of therapy, depending on the type of infection?16. Does the app support the user in adjusting medications based on the patient’s kidney function?17. Does the app support the user in adjusting the dosage/medications to be prescribed in the case of a pediatric patient?18. Does the app provide information about possible side effects and allergic reactions to these medications?19. Does the app provide useful information for prescribing alternative medications if a patient is allergic to first-line medications?20. Does the app indicate the safety class of the medication for use during pregnancy?21. Does the app provide information about antifungal therapies?22. Does the app support the user in correctly reading an antibiogram?23. Does the app support the user in identifying the drug for targeted therapy against a specific microorganism?24. Does the app support the user in switching from empiric therapy to targeted therapy?25. Does the app enable information about the dosage to be prescribed?26. Does the app consider clinical variables related to the location of the infection and patient characteristics when choosing therapy?27. Does the app indicate the range of prescription of the drug (range A or range C or hospital regimen)?
Antimicrobial resistance (AMR)	28. Does the app provide information on the developmental mechanism and types of antibiotic resistance (eg, plasmid, chromosomal)?29. Does the app provide data on the prevalence of antimicrobial resistance worldwide?30. Does the app provide data on the prevalence of antimicrobial resistance phenomena at the European level?31. Does the app provide data on the prevalence of antimicrobial resistance phenomena at the Italian level?32. Does the app provide data on the prevalence of antimicrobial resistance phenomena at the regional level?33. Does the app report the need to isolate the patient?
Dashboard function	34. Does the app provide up-to-date global/worldwide data on antimicrobial resistance profiles of isolates?35. Does the app report updated European-level data on antimicrobial resistance profiles of isolates?36. Does the app report updated data on the Italian-level on isolate antimicrobial resistance?37. Does the app report regionally updated data on the antimicrobial resistance profiles of isolates?
Antimicrobial stewardship (AMS)	38. Does the app provide information on the latest available guidelines?39. Do the guidelines or indications included in the app differentiate between prophylaxis and therapy?40. Does the app describe the use of antibiotics in prophylaxis (eg, antibiotic therapy in neutropenic leukemic patients, endocarditis prophylaxis)?41. Does the app include information about the circulation of the same pathogen between human-animal-environment?42. Does the app inform the user about the role of the veterinary component in AMR/AMS from a One Health perspective?43. Does the app inform the user about the role of the environmental component in AMR/AMS from a One Health perspective?
Notes and records	44. Does the app allow the user to enter data for an antimicrobial resistance database (eg, lack of response to therapy)?45. Does the app allow users to store notes or personal information?46. Does the app allow users uploading of clinical patient data?47. Does the app allow users to store individua therapy regimens (eg, clinical trials, innovative therapy associations)?48. Does the app provide warnings about potential unsafe uses of a particular therapy associated with specific clinical conditions?49. Does the app provide warnings about potential drug-drug or drug-food interactions?
Network	50. Does the app provide mechanisms for users to interact with other users?51. Does the app provide references and contacts of a service that prescribers can contact with clinical questions?52. Does the app provide different levels of interaction between users (eg, between peers, different professional profiles)?53. Does the app provide different levels of interaction that allow consultation with experts, pharmacologists, microbiologists, etc?54. Does the app provide other social mechanisms for users to share experiences?55. Is the app connected to the Italian post-marketing surveillance network (vigifarmaco) for reporting adverse effects?56. Is the app connected to the infectious disease reporting system?
Technical features	57. Does the app ask users for authentication?58. Does the app have a privacy policy?59. Are all app contents freely available to the users (without any payment)?60. Are there specific inclusion criteria for full app usage (eg, authorization from a company/region/professional order)?61. Does the app require a workplace as an inclusion criterion for its full use?62. Does the app allow for preselection of treatment setting (primary care/long-term care facilities/nursing home/hospital)?63. Does the app require to “sign” an informed consent for app usage?64. Does the app include a glossary of the most used terms/abbreviations?65. Does the app identify the scientific responsibility of the provided contents?66. Is there a possibility to back-up/restore data within the app?67. Is there a possibility to download data collected through the app?68. Does the app have multilanguage support?69. Does the app interact with the hospital or local medical management software?70. Does the app geolocate the user to provide more detailed information?71. Does the app provide a real-time map of the AMR in relation to the country of reference (even if it is not the usual location of the practice)?72. Does the app allow users to update their account preferences?73. Does the app adapt to screen orientation (both portrait and landscape)?74. Does the app learn user’s preferences over time?75. Does the app implement intuitive navigation patterns?76. Does the app implement predictable navigation patterns?77. Has the app content been validated by a local institutional source?78. Has the app content been validated by a regional institutional source?79. Has the app content been validated by a national institutional source?80. Has the app content been validated by a global institutional source?81. Is the app a certified medical device according to Italian law?82. Does the app provide content in text mode?83. Does the app provide content in audio mode?84. Does the app provide content in video mode?85. Can the app be used offline in its entirety?86. Does the app offer a technical support center to contact?

### Ethical Considerations

This study did not include human subjects research (no human subjects experimentation or intervention was conducted) and so did not require institutional review board approval.

## Results

### Step 1: Systematic Search

**Figure 1.**
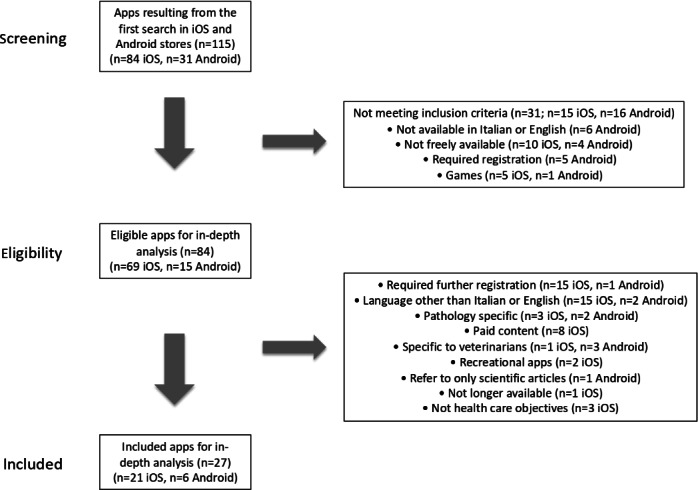
Results of the app selection process.

First, 115 apps were identified: 31 apps for Android and 84 apps for iOS. By applying the exclusion criteria, 31 apps were excluded: 16 for Android and 15 for iOS. The remaining 84 eligible apps (15 for Android and 69 for iOS) were downloaded, installed, and further analyzed using the same criteria, excluding 57 apps: 48 for iOS and 9 for Android. The remaining 27 apps (6 for Android and 21 for iOS) were selected for in-depth analysis. Based on additional inclusion and exclusion criteria, as outlined in Figure 1, the flowchart illustrates the app selection process and outcomes.

### Step 2: App Evaluation

After the entire selection process, 27 apps were evaluated using the checklist questions, of which 6 were available for Android (22%), 21 (78%) were available for iOS, and 1 was available in both stores. In total, 12 of the 27 apps were associated or affiliated with health care institutions or other international organizations. As shown in Table 2, considering all the potential items that could be fulfilled by the apps for each domain, of the 27 apps selected, diagnosis and therapy support (90/513, 37%) and app technical characteristics (187/810, 23%) were the most frequently fulfilled domains, followed by AMS (13/162, 8%), pathogens and etiological agents (8/216, 4%), notes and records (5/162, 3%), network (4/189, 2%), AMR (2/162, 1%), and dashboard function (1/108, 1%).

In the domain of pathogens and etiological agents, only 5 apps (19%) provided information on the mechanism of action of drugs, 2 apps (7%) provided information on the main characteristics of microorganisms, and 1 app provided information on the characteristics of aerobic and anaerobic microorganisms. In the domain of diagnostic and therapeutic support, 21 (78%) apps assisted the user in identifying the drug for targeted therapy against a specific microorganism, 18 (67%) apps provided information on the dosage to be prescribed, 17 apps (63%) indicated the route of administration for the drugs indicated for therapy, and 15 apps (56%) assisted the user in selecting the drug for empirical therapy according to the patient’s clinical condition, and 15 apps (56%) considered the clinical variables related to the site of infection and patient characteristics in their therapy suggestions. In contrast, most apps (n=23, 85%) did not support the user in reading the antibiogram correctly. In the domain of AMR, hardly any app fulfilled the required characteristics, with the exception of one app that provided information on the evolution and types of AMR and one app that provided prevalence data on AMR in Italy, while no app provided prevalence data at European and Italian level.

In terms of dashboard functions, only 1 app responded to one of the items by providing regional data on AMR profiles of isolates; national and international data were not provided to the user. In relation to AMS, 8 apps (30%) provided information on the latest available guidelines, but little other information was provided on this topic. In particular, no app provided information on the possible circulation of the same pathogen between humans, animals, and the environment and did not inform the user about the role of the veterinary component in AMR and AMS from a One Health perspective. In terms of the network domain, no app was connected to the Italian postmarketing surveillance network for reporting possible adverse effects or to the infectious disease reporting system. No app offered opportunities to interact with other users or experts.

Regarding technical features, all apps (100%) had intuitive and predictable navigation patterns and offered content in text mode, the content of most apps (26/27, 96%) was available to users without payment, and 81% of the apps (22/27) could be used completely offline. However, none of the apps provided a real-time map of the AMR in relation to the reference country or located the user to provide more detailed information. In addition, none of the apps interacted with the hospital or local medical management software, nor did they offer backup, restore, or download functions. In addition, no app offered multilingual support or was certified as a medical device under Italian law, and only 11 apps (41%) claimed scientific responsibility for the content provided. Multimedia Appendix 1 shows the detailed results of the app analysis.

Multimedia Appendix 2 shows the detailed results of the in-depth analysis of each of the 27 apps.

Considering the full list of items, no app met all the desirable criteria and the highest level of fulfilment of the desirable items identified by the checklist was 36%, which was only achieved by 2 apps. All other apps achieved fulfilment rates between 9% and 26%.

**Table 2.** Level of fulfillment for each domain.

	Information, functionality, and features provided by the app		
Domain^a^	Yes, n (%)	No, n (%)	Partially, n (%)
Pathogens and etiological agents (n=216)	8 (4)	208 (96)	0 (0)
Diagnostic and therapeutic support (n=513)	190 (37)	320 (62)	3 (1)
Antimicrobial resistance (n=162)	2 (1)	160 (99)	0 (0)
Dashboard function (n=108)	1 (1)	107 (99)	0 (0)
Antimicrobial stewardship (n=162)	13 (8)	149 (92)	0 (0)
Notes and records (n=162)	5 (3)	156 (96)	1(1)
Network (n=189)	4 (2)	185 (98)	0 (0)
Technical features (n=810)	187 (23)	623 (77)	0 (0)

a In brackets are reported the total number of items evaluated for accomplishment for each domain on the total of 27 apps.

## Discussion

The aim of this study was to search and evaluate the mHealth apps available in Italy to support health care professionals and, in particular, prescribers in the fight against AMR.

### Principal Findings

Most of the apps analyzed did not have most of the desirable features, with only two of them achieving a 36% fulfilment level. Most of these apps lacked the provision of content on pathogen characteristics, diagnostic and therapeutic support, AMR, and AMS. The dashboard function was generally lacking and did not allow users to view data on local, national, and international AMR prevalence. The mHealth solutions did not support a network system, which meant that organizational and technical support between professionals could not be provided. One of the most likely reasons why these apps did not meet the criteria selected by the expert group could be that they were designed and developed for other purposes and were not aimed at supporting health care professionals in AMS. Not having a dashboard function is missing an opportunity both at the national and international level, because having local data available could be useful to prescribers.

We are aware that we identified many features and that probably no app could have responded positively to all the items. Nevertheless, to reach our purpose to fighting AMR, we inserted all the elements we considered useful for the good functioning of an app in order to verify the distance between apps available for free and our ideal standards. Starting from this study, the next step we are planning is to create an app that fulfills most of our items.

We chose to provide data about the evaluation of each of the 27 apps in Multimedia Appendix 1. This makes it possible for readers to analyze apps also using subgroups of items. Ensuring an audit and feedback mechanism for prescribers by bringing together relevant information can indeed promote changes in practice, as is done in many clinical contexts [21] through quality improvement programs [22]. Therefore, the audit and feedback mechanism for prescribers and the opportunity for benchmarking should be supported to enhance their role in practice [23]. In addition, the recent pandemic has revolutionized the previous approach of health care professionals and researchers seeking access to organizational and institutional sources of information. This process toward greater transparency, accountability, and democratization of health data began with the introduction of the free availability of Italian COVID-19 surveillance data in 2020 [24]. The use of technology is not only changing the way people and professionals communicate with each other, but is also providing innovative ways to monitor the health and well-being of patients and the planet, and giving health care professionals better access to information for a more holistic view of health issues. Digital technologies are emerging as an important resource for health care and public health [15], but much remains to be done to translate evidence-based, validated information into clinical practice through medically certified devices. Probably the most recent, scientifically pervasive, and currently ground-breaking topic that is receiving massive and increasing investment in the public sector, but even more so in the private sector, is AI. In health care, AI is already being used to some extent for chatbots (software applications or web interfaces, designed to have textual or spoken conversations) and triage systems to assess the need for doctor visits. It is also sometimes used in diagnostics, where it achieves accuracy comparable to that of experts, in predictive modelling, pharmaceutical research, and epidemiological studies [25]. As for its use in antimicrobial devices, ChatGPT (a comprehensive language model developed by OpenAI) appears to have access to sufficient training data (although it does not have access to specific medical databases), but has shortcomings in consistency, reasoning, and situational awareness that could jeopardize patient health. missing important aspects in scenarios of increasing complexity [26]. Despite these advances, problems remain regarding the robustness of studies and algorithmic bias. Furthermore, there are currently no specific regulations for AI-assisted care. If AI systems prove to be sufficiently reliable, they could provide significant support to health care providers. Although the use of AI in clinical practice is still limited, initial studies show promising results, suggesting that external validation and clinical trials are likely to increase in the near future [27]. Most importantly, local, regional, and national institutions and professionals need real-time data at the local, regional, and national level to make the best clinical and organizational decisions regarding antimicrobial stewardship. Therefore, the availability of features to train algorithms with real AMR and AMS data without the risk of data breaches is critical to the pursuit of this goal.

The Italian National Plan to Combat Antimicrobial Resistance (Piano Nazionale di Contrasto dell’Antimicrobico-Resistenza, PNCAR) recommends a One Health approach to contain AMR [12]. The apps analyzed in this study lack information on the circulation of zoonotic AMR pathogens between humans and animals and on the role of the veterinary component and the environment in the AMR and AMS problem, thus missing the opportunity to consider the broader One Health perspective. To effectively contribute to global health security by supporting prevention, early detection and warning, response and recovery for emerging and endemic communicable diseases, the One Health approach requires cross-sectoral integration and collaboration to efficiently and effectively bring together information, capacity and expertise to protect people, animals and our environment. On this basis, the tripartite collaboration between the Food and Agriculture Organization (FAO), the World Organisation for Animal Health (OIE) and the WHO has recently reaffirmed its commitment to cross-sectoral collaboration on disease risks at the human-animal-environment interface [28], which is also supported by many other global and local stakeholders [29]. Given the high economic risks of disease epidemics and their far-reaching societal and economic impacts, cross-sectoral One Health approaches are critical to improving prevention, detection, response, and recovery capacity [30]. In line with this vision, any action to address climate change can in turn benefit the global burden of disease, migration, conflict over natural resources and political instability—all of which are closely linked to the economic, environmental, and social determinants of health. In Europe, for example, climate change has been observed to lead to changes in rates of communicable and noncommunicable diseases due to temperature changes, increase the risk of outbreaks and exacerbations of clinical diseases (especially cardiovascular diseases), influence extreme meteorological events (eg, rainfall, floods, and hurricanes), cause sea level rise, and alter the distribution of plant species and arthropod vectors, as well as the quality of air and food. Nonetheless, climate change also has an impact on food security, as an increase in global temperature is associated with an increase in global demand for food [31].

Measures to improve the appropriateness of antimicrobial prescribing are a key component of AMS programs [10,32], as it is widely recognized that inappropriate use of antimicrobials increases the risk of treatment failures and adverse events in individual patients and accelerates the selection and transmission of antimicrobial-resistant pathogens in health care facilities and populations [33]. Nonetheless, the awareness and knowledge of prescribing physicians on these issues still needs to be improved and strengthened. To achieve this goal, much needs to be done, from preparation to ongoing education of health care professionals, to reinforce the message and support the behaviors. To promote a culture of optimal antibiotic use, the WHO published guidance in 2022 based on the AWaRe classification, which was introduced to support measures to improve antibiotic management. Antibiotics are categorized into 3 groups (Aware, Watch, and Reserve) according to their clinical importance and the risk that their use promotes the development of resistance. The guidelines contain evidence-based information on the selection, formulation, dosing, and duration of administration of antibiotics and defines clinical situations in which antibiotics are not recommended based on the best scientific evidence [34]. The WHO’s “Firstline” app, which is only partially available to unregistered users, is also moving in this direction. This app provides various national and some local guidelines on different types of infections and some of their antibiotic treatment for adults and children, and its content is also available online [35]. In Italy, for example, “Firstline” is temporarily available for the pilot phase of the Enforcing Surveillance of Antimicrobial Resistance and Antibiotic Use to Drive Stewardship (ENSURE project) in Padua or for the users of the Integrated University Hospital of Verona after authentication. However, as this is a first attempt to address the problem, more advanced solutions are being sought to match guidelines with data and prescribing behavior to reverse the general trend. Antibiogo (Médecins Sans Frontières Foundation) is a mobile app, which was developed to help laboratory workers without expertise in low-resource settings measure and evaluate antibiotic susceptibility tests so that clinicians can prescribe the right antibiotics to their patients [36].

The European Centre for Disease Prevention and Control (ECDC) has defined core competencies for professionals in the field of infection control and hospital hygiene. These include minimum requirements common to all professionals in this field, ranging from leadership and management of infection prevention and control programs, microbiology and surveillance, infection prevention and control in clinical practice, education and training, quality, patient safety and occupational health [37]. Another issue to consider in relation to this topic is the sale of over-the-counter (OTC) antibiotics. This is seen as a significant problem, particularly in regions of the world (ie, low- and middle-income countries) where prevalence is quite high. In these areas, it is not enough just to influence prescribers to tackle the problem of antimicrobial resistance; other measures must also be taken to control the use of antimicrobials. For this reason, the WHO has launched an electronic consultation in 2023 involving key stakeholders such as politicians, pharmacist associations, and civil society to identify possible actions for a global campaign to phase out over-the-counter antibiotics [38]. Despite these considerations and the awareness that there is still much to be done in this area, we believe that this study was able to provide an initial insight into the information that already exists and what could be done to further support health care professionals in combating AMR. For example, providing an institutionally validated app with content tailored to the individual needs of the health care professional and setting would help to tailor prescribing decisions to the context and tailor support to the prescriber’s expertise. For example, the app could differentiate between primary care providers (general practitioners, family medicine, pediatrics, and internal medicine), acute care providers (inpatient or hospitalists), and infectious disease specialists to provide the most needed information.

### Limitations

This study has several limitations. First, the exclusion criteria of this study limited the systematic search for the apps available in the Apple App Store and Google Play Store, specifically the requirement that the app must be available for free and without registration to be included in the in-depth analysis. These criteria aimed to evaluate the apps available to all health care professionals, regardless of their geographical or institutional affiliation. During the screening phase and the eligibility phase excluded apps were overall 22 of 115 (19%). Although aware that this share is relevant, we opted to exclude them anyway, because of the consideration that was more important that the contents were freely available to health care professionals, without the obligation of payment, in order to ensure equity for all economic levels. However, by choosing to include both Italian- and English-language apps, apps were selected that primarily target the educational level and professional profile of prescribing physicians. Second, we cannot know whether presenting the same checklist to other larger groups of experts would confirm all the items on the checklist we used to test the apps. Therefore, further future research along these lines may be useful to confirm, model, refine, and integrate domains and items. Third, the availability of the apps in stores at the time of the systematic search and a limited app validation panel may have limited this study. The search strategy, which involved the use of keywords, was probably unable to find some apps containing AMR modules. However, our goal was to find an app totally and not partially focused on AMR. Furthermore, this study does not value the length of therapy based on the organism and site of infection, surely an AMS domain. It could be a parameter to include in future works.

Finally, the actual effectiveness of the apps was not evaluated as this was not our focus. Nonetheless, this initial evaluation could form the basis for future work exploring this aspect in more depth.

### Conclusions

This systematic search for apps available to Italian health care professionals to support antimicrobial prescribing in line with AMS principles shows that there is still much room for improvement in the development of apps and more generally in mHealth solutions to combat antimicrobial resistance. There is currently no user-friendly and useful app that meets all the requirements identified by the expert group; such a tool would support the engagement of prescribers in the fight against AMR.

## Supplementary material

10.2196/51122 Multimedia Appendix 1 Total number and percentage of apps meeting the items.

10.2196/51122 Multimedia Appendix 2 Total number and percentage of accomplishments for each questionnaire item and globally for each of the 27 apps.

## Abbreviations

AI: artificial intelligence
AMR: antimicrobial resistance
AMS: antimicrobial stewardship
ECDC: European Centre for Disease Prevention and Control
EEA: European Economic Area
EHR: electronic health record
ENSURE: Enforcing Surveillance of Antimicrobial Resistance and Antibiotic Use to Drive Stewardship
EU: European Union
FAO: Food and Agriculture Organization
mHealth: mobile health
OTC: over-the-counter
PNCAR: Piano Nazionale di Contrasto dell’Antimicrobico-Resistenza
SDG: Sustainable Development Goal
WHO: World Health Organization
