# Supplementary material for: mHealth Apps Available in Italy to Support Health Care Professionals in Antimicrobial Stewardship Implementation: Systematic Search in App Stores and Content Analysis
Source: JMIR Mhealth Uhealth. 2025 Apr 29;13:e51122. doi: 10.2196/51122 (PMC12054965; doi:10.2196/51122)
Supplement: Multimedia Appendix 1 [file mhealth-v13-e51122-s001.docx]

Table 3. Total number and percentage of apps meeting the items.

| Domain | Item | Tot yes (n; %) | Tot  no  (n; %) | Tot partially (n; %) |
| --- | --- | --- | --- | --- |
| Pathogens/etiological agents | Does the app contain information about the main microbiological characteristics of microorganisms? | 2; 7 | 25; 93 | 0; 0 |
|  | Does the app contain information about the characteristics of aerobic and anaerobic microorganisms? | 1; 4 | 26; 97 | 0; 0 |
|  | Does the app contain information about the cell wall of microorganism? | 0; 0 | 27; 100 | 0; 0 |
|  | Does the app contain information about the main characteristics of fungal pathogens? | 0; 0 | 27; 100 | 0; 0 |
|  | Does the app contain information about the availability of vaccines that can prevent infections/diseases caused by infectious agents? | 0; 0 | 27; 100 | 0; 0 |
|  | Does the app include information about the methods and timeline for reporting infectious diseases? | 0; 0 | 27; 100 | 0; 0 |
|  | Does the app provide information on how antimicrobial drugs work? | 5; 19 | 22; 81 | 0; 0 |
|  | Does the app provide guidance on infections prevention and control (PPE, procedures)? | 0; 0 | 27; 100 | 0; 0 |
| Diagnostic and therapeutic support | Does the app contain information about infections and their aetiology? | 10; 37 | 17; 63 | 0; 0 |
|  | Does the app include information to help distinguish between a community-acquired infection (CAI) and a healthcare-acquired infection (HAI)? | 4; 15 | 23; 85 | 0; 0 |
|  | Does the app include references to diagnostic tests that are useful in formulating the diagnosis? | 5; 19 | 22; 81 | 0; 0 |
|  | Does the app support the user in selecting a drug for empiric therapy according to the clinical condition? | 15; 56 | 12; 44 | 0; 0 |
|  | Does the app indicate the route of administration of the medications indicated for therapy? | 17; 63 | 10; 37 | 0; 0 |
|  | Does the app provide specific information on drug pharmacokinetics and pharmacodynamics? | 3; 11 | 24; 89 | 0; 0 |
|  | Does the app indicate the expected duration of therapy, depending on the type of infection? | 12; 44 | 14; 52 | 1; 4 |
|  | Does the app support the user in adjusting medications based on the patient’s kidney function? | 6; 22 | 21; 78 | 0; 0 |
|  | Does the app support the user in adjusting the dosage/medications to be prescribed in the case of a paediatric patient? | 12; 44 | 15; 56 | 0; 0 |
|  | Does the app provide information about possible side effects and allergic reactions to these medications? | 7; 26 | 18; 67 | 2; 7 |
|  | Does the app provide useful information for prescribing alternative medications if a patient is allergic to first-line medications? | 14; 52 | 13; 48 | 0; 0 |
|  | Does the app indicate the safety class of the medication for use during pregnancy? | 3; 11 | 24; 89 | 0; 0 |
|  | Does the app provide information about antifungal therapies? | 10; 37 | 17; 63 | 0; 0 |
|  | Does the app support the user in correctly reading an antibiogram? | 4; 15 | 23; 85 | 0; 0 |
|  | Does the app support the user in identifying the drug for targeted therapy against a specific microorganism? | 21; 78 | 6; 22 | 0; 0 |
|  | Does the app support the user in switching from empiric therapy to targeted therapy? | 13; 48 | 14; 52 | 0; 0 |
|  | Does the app enable information about the dosage to be prescribed? | 18; 67 | 9; 33 | 0; 0 |
|  | Does the app consider clinical variables related to the location of the infection and patient characteristics when choosing therapy? | 15; 56 | 12; 44 | 0; 0 |
|  | Does the app indicate the range of prescription of the drug (range A/ range C/ hospital regimen)? | 1; 4 | 26; 96 | 0; 0 |
| Antimicrobial resistance (AMR) | Does the app provide information on the developmental mechanism and types of antibiotic resistance (e.g., plasmid, chromosomal)? | 1; 4 | 26; 96 | 0; 0 |
|  | Does the app provide data on the prevalence of antimicrobial resistance worldwide? | 0; 0 | 27; 100 | 0; 0 |
|  | Does the app provide data on the prevalence of antimicrobial resistance phenomena at the European level? | 0; 0 | 27; 100 | 0; 0 |
|  | Does the app provide data on the prevalence of antimicrobial resistance phenomena at the Italian level? | 0; 0 | 27; 100 | 0; 0 |
|  | Does the app provide data on the prevalence of antimicrobial resistance phenomena at the regional level? | 1; 4 | 26; 96 | 0; 0 |
|  | Does the app report the need to isolate the patient? | 0; 0 | 27; 100 | 0; 0 |
| Dashboard function | Does the app provide up-to-date global/worldwide data on antimicrobial resistance profiles of isolates? | 0; 0 | 27; 100 | 0; 0 |
|  | Does the app report updated European-level data on antimicrobial resistance profiles of isolates? | 0; 0 | 27; 100 | 0; 0 |
|  | Does the app report updated data on the Italian-level on isolate antimicrobial resistance? | 0; 0 | 27; 100 | 0; 0 |
|  | Does the app report regionally updated data on the antimicrobial resistance profiles of isolates? | 1; 4 | 26; 96 | 0; 0 |
| Antimicrobial stewardship (AMS) | Does the app provide information on the latest available guidelines? | 8; 30 | 19; 70 | 0; 0 |
|  | Do the guidelines or indications included in the app differentiate between prophylaxis and therapy? | 3; 11 | 24; 89 | 0; 0 |
|  | Does the app describe the use of antibiotics in prophylaxis (e.g., antibiotic therapy in neutropenic leukemic patients, endocarditis prophylaxis)? | 2; 7 | 25; 93 | 0; 0 |
|  | Does the app include information about the circulation of the same pathogen between human-animal-environment? | 0; 0 | 27; 100 | 0; 0 |
|  | Does the app inform the user about the role of the veterinary component in AMR/AMS from a One Health perspective? | 0; 0 | 27; 100 | 0; 0 |
|  | Does the app inform the user about the role of the environmental component in AMR/AMS from a One Health perspective? | 0; 0 | 27; 100 | 0; 0 |
| Notes and records | Does the app allow the user to enter data for an antimicrobial resistance database (e.g., lack of response to therapy)? | 0; 0 | 27; 100 | 0; 0 |
|  | Does the app allow users to store notes or personal information? | 2; 7 | 25; 93 | 0; 0 |
|  | Does the app allow users uploading of clinical patient data? | 0; 0 | 27; 100 | 0; 0 |
|  | Does the app allow users to store individual therapy regimens (e.g., clinical trials, innovative therapy associations)? | 0; 0 | 27; 100 | 0; 0 |
|  | Does the app provide warnings about potential unsafe uses of a particular therapy associated with specific clinical conditions? | 1; 4 | 26; 96 | 0; 0 |
|  | Does the app provide warnings about potential drug-drug or drug-food interactions? | 2; 7 | 24; 89 | 1; 4 |
| Network | Does the app provide mechanisms for users to interact with other users? | 0; 0 | 27; 100 | 0; 0 |
|  | Does the app provide references and contacts of a service that prescribers can contact with clinical questions? | 4; 15 | 23; 85 | 0; 0 |
|  | Does the app provide different levels of interaction between users (e.g., between peers, different professional profiles)? | 0; 0 | 27; 100 | 0; 0 |
|  | Does the app provide different levels of interaction that allow consultation with experts, pharmacologists, microbiologists, etc.? | 0; 0 | 27; 100 | 0; 0 |
|  | Does the app provide other social mechanisms for users to share experiences? | 0; 0 | 27; 100 | 0; 0 |
|  | Is the app connected to the Italian post-marketing surveillance network (vigifarmaco) for reporting adverse effects? | 0; 0 | 27; 100 | 0; 0 |
|  | Is the app connected to the infectious disease reporting system? | 0; 0 | 27; 100 | 0; 0 |
| Technical features | Does the app ask users for authentication? | 1; 4 | 26; 96 | 0; 0 |
|  | Does the app have a privacy policy? | 19; 70 | 8; 30 | 0; 0 |
|  | Are all app contents freely available to the users (without any payment)? | 26; 96 | 1; 4 | 0; 0 |
|  | Are there specific inclusion criteria for full app usage (e.g., authorization from a company/region/professional order)? | 0; 0 | 27; 100 | 0; 0 |
|  | Does the app require a workplace as an inclusion criterion for its full use? | 0; 0 | 27; 100 | 0; 0 |
|  | Does the app allow for pre-selection of treatment setting (primary care/long-term care facilities/nursing home/hospital)? | 0; 0 | 27; 100 | 0; 0 |
|  | Does the app require to “sign” an informed consent for app usage? | 0; 0 | 27; 100 | 0; 0 |
|  | Does the app include a glossary of the most used terms/abbreviations? | 2; 7 | 25; 93 | 0; 0 |
|  | Does the app identify the scientific responsibility of the provided contents? | 11; 41 | 16; 59 | 0; 0 |
|  | Is there a possibility to back-up/restore data within the app? | 0; 0 | 27; 100 | 0; 0 |
|  | Is there a possibility to download data collected through the app? | 0; 0 | 27; 100 | 0; 0 |
|  | Does the app have multilanguage support? | 0; 0 | 27; 100 | 0; 0 |
|  | Does the app interact with the hospital or local medical management software? | 0; 0 | 27; 100 | 0; 0 |
|  | Does the app geolocate the user to provide more detailed information? | 0; 0 | 27; 100 | 0; 0 |
|  | Does the app provide a real-time map of the AMR in relation to the country of reference (even if it is not the usual location of the practice)? | 0; 0 | 27; 100 | 0; 0 |
|  | Does the app allow users to update their account preferences? | 0; 0 | 27; 100 | 0; 0 |
|  | Does the app adapt to screen orientation (both portrait and landscape)? | 15; 56 | 12; 44 | 0; 0 |
|  | Does the app learn user’s preferences over time? | 0; 0 | 27; 100 | 0; 0 |
|  | Does the app implement intuitive navigation patterns? | 27; 100 | 0; 0 | 0; 0 |
|  | Does the app implement predictable navigation patterns? | 27; 100 | 0; 0 | 0; 0 |
|  | Has the app content been validated by a local institutional source? | 4; 15 | 23; 85 | 0; 0 |
|  | Has the app content been validated by a regional institutional source? | 1; 4 | 26; 96 | 0; 0 |
|  | Has the app content been validated by a national institutional source? | 2; 7 | 25; 93 | 0; 0 |
|  | Has the app content been validated by a global institutional source? | 0; 0 | 27; 100 | 0; 0 |
|  | Is the app a certified medical device according to Italian law? | 0; 0 | 27; 100 | 0; 0 |
|  | Does the app provide content in text mode? | 27; 100 | 0; 0 | 0; 0 |
|  | Does the app provide content in audio mode? | 0; 0 | 27; 100 | 0; 0 |
|  | Does the app provide content in video mode? | 0; 0 | 27; 100 | 0; 0 |
|  | Can the app be used offline in its entirety? | 22; 81 | 5; 19 | 0; 0 |
|  | Does the app offer a technical support center to contact? | 3; 11 | 24; 89 | 0; 0 |
