# Supplementary material for: mHealth Apps Available in Italy to Support Health Care Professionals in Antimicrobial Stewardship Implementation: Systematic Search in App Stores and Content Analysis
Source: JMIR Mhealth Uhealth. 2025 Apr 29;13:e51122. doi: 10.2196/51122 (PMC12054965; doi:10.2196/51122)
Supplement: Multimedia Appendix 2 [file mhealth-v13-e51122-s002.docx]

| Domain | Item | Pharmapedia – free medicine Encyclopedia | CliMic | Infect by Anresis | Prescribing Companion App | Bugs & Drugs 2.0 | Sepsis Clinical Guide | GP Antibiotics(iOS) | Antibiotics Essentials | Pocket spectrum-antibiotics | AIIMS Antibiotic Policy | Antibiotics for common infects | Antibiotics Herts & Wessex ICS | BLMK Antibiotics | Impact app | K-APP | Drug Explorer | Vanuatu Guideline Host | Antimicrobial Companion | Antibiograms | BugBuster 3000 | Bluebook/NAG KKM Malaysia | Antibiotics | Antibiotics flashcards | Antibiotic of common infection | Antibiotics | AMR Treatment Guidelines 2022 | GP Antibiotics (Android) | Tot y  (n;%) | Tot n  (n;%) | Tot p  (n;%) |  |
| --- | --- | --- | --- | --- | --- | --- | --- | --- | --- | --- | --- | --- | --- | --- | --- | --- | --- | --- | --- | --- | --- | --- | --- | --- | --- | --- | --- | --- | --- | --- | --- | --- |
| Pathogens/  etiological agents | 1 | n | n | n | n | y | n | n | n | n | y | n | n | n | n | n | n | n | n | n | n | n | n | n | n | n | n | n | 2;7 | 25;93 | 0;0 |  |
|  | 2 | n | n | n | n | y | n | n | n | n | n | n | n | n | n | n | n | n | n | n | n | n | n | n | n | n | n | n | 1;4 | 26;97 | 0;0 |  |
|  | 3 | n | n | n | n | n | n | n | n | n | n | n | n | n | n | n | n | n | n | n | n | n | n | n | n | n | n | n | 0;0 | 27;100 | 0;0 |  |
|  | 4 | n | n | n | n | n | n | n | n | n | n | n | n | n | n | n | n | n | n | n | n | n | n | n | n | n | n | n | 0;0 | 27;100 | 0;0 |  |
|  | 5 | n | n | n | n | n | n | n | n | n | n | n | n | n | n | n | n | n | n | n | n | n | n | n | n | n | n | n | 0;0 | 27;100 | 0;0 |  |
|  | 6 | n | n | n | n | n | n | n | n | n | n | n | n | n | n | n | n | n | n | n | n | n | n | n | n | n | n | n | 0;0 | 27;100 | 0;0 |  |
|  | 7 | y | n | n | n | n | n | n | n | n | y | n | n | n | n | n | n | n | n | n | n | n | y | y | n | y | n | n | 5;19 | 22;81 | 0;0 |  |
|  | 8 | n | n | n | n | n | n | n | n | n | n | n | n | n | n | n | n | n | n | n | n | n | n | n | n | n | n | n | 0;0 | 27;100 | 0;0 |  |
| Diagnostic and therapeutic support | 9 | n | n | n | n | y | n | n | n | n | y | y | y | y | y | y | n | n | y | n | n | y | n | n | y | n | n | n | 10;37 | 17;63 | 0;0 |  |
|  | 10 | n | n | n | n | n | n | n | n | n | y | n | n | n | y | n | n | n | y | y | n | n | n | n | n | n | n | n | 4;15 | 23;85 | 0;0 |  |
|  | 11 | n | n | y | n | y | n | n | n | n | y | n | n | n | y | n | n | n | n | n | y | n | n | n | n | n | n | n | 5;19 | 22;81 | 0;0 |  |
|  | 12 | n | n | y | n | y | n | y | y | n | y | y | y | y | y | y | y | n | y | n | y | y | n | n | n | n | y | n | 15;56 | 12;44 | 0;0 |  |
|  | 13 | n | y | y | n | y | y | n | y | n | y | n | y | y | y | y | n | n | y | n | y | y | y | y | y | n | n | y | 17;63 | 10;37 | 0;0 |  |
|  | 14 | n | n | n | n | y | n | n | y | n | n | n | n | n | n | n | n | n | n | n | n | n | y | n | n | n | n | n | 3;11 | 24;89 | 0;0 |  |
|  | 15 | n | n | y | n | y | n | y | y | n | y | y | y | y | n | y | n | n | n | n | y | n | n | n | y | n | y | p | 12;44 | 14;52 | 1;4 |  |
|  | 16 | n | n | n | n | y | y | n | n | n | y | n | n | n | n | y | y | n | n | n | y | n | n | n | n | n | n | n | 6;22 | 21;78 | 0;0 |  |
|  | 17 | n | n | y | n | y | y | y | n | n | n | n | y | y | n | y | y | n | n | n | y | y | n | n | y | n | n | y | 12;44 | 15;56 | 0;0 |  |
|  | 18 | y | n | n | n | y | n | y | n | n | n | n | n | n | p | p | n | n | n | n | n | n | y | y | y | y | n | n | 7;26 | 18;67 | 2;7 |  |
|  | 19 | n | n | y | n | y | n | y | n | n | y | y | n | y | y | n | n | n | y | y | y | n | n | y | y | n | y | y | 14;52 | 13;48 | 0;0 |  |
|  | 20 | n | n | n | n | y | n | n | n | n | n | n | n | y | n | y | n | n | n | n | n | n | n | n | n | n | n | n | 3;11 | 24;89 | 0;0 |  |
|  | 21 | n | n | n | n | y | y | y | n | y | y | n | y | y | n | y | n | n | n | n | y | y | n | n | n | n | n | n | 10;37 | 17;63 | 0;0 |  |
|  | 22 | n | n | y | n | y | n | n | n | n | n | n | n | n | y | n | n | n | n | y | n | n | n | n | n | n | n | n | 4;15 | 23;85 | 0;0 |  |
|  | 23 | n | y | y | n | y | n | y | n | y | y | y | y | y | y | y | y | n | y | y | y | y | y | y | y | y | n | y | 21;78 | 6;22 | 0;0 |  |
|  | 24 | n | n | y | n | y | n | y | n | n | y | y | y | y | y | y | y | n | y | n | y | y | n | n | n | n | n | n | 13;48 | 14;52 | 0;0 |  |
|  | 25 | n | n | y | n | y | y | y | y | n | y | y | y | y | y | y | y | y | y | n | y | y | y | y | y | n | n | y | 18;67 | 9;33 | 0;0 |  |
|  | 26 | n | n | y | n | y | y | y | y | n | y | y | y | y | y | y | y | n | y | n | y | y | n | n | n | n | n | n | 15;56 | 12;44 | 0;0 |  |
|  | 27 | n | n | n | n | y | n | n | n | n | n | n | n | n | n | n | n | n | n | n | n | n | n | n | n | n | n | n | 1;4 | 26;96 | 0;0 |  |
| Antimicrobial resistance (AMR) | 28 | n | n | n | n | n | n | n | n | n | n | n | n | n | y | n | n | n | n | n | n | n | n | n | n | n | n | n | 1;4 | 26;96 | 0;0 |  |
|  | 29 | n | n | n | n | n | n | n | n | n | n | n | n | n | n | n | n | n | n | n | n | n | n | n | n | n | n | n | 0;0 | 27;100 | 0;0 |  |
|  | 30 | n | n | n | n | n | n | n | n | n | n | n | n | n | n | n | n | n | n | n | n | n | n | n | n | n | n | n | 0;0 | 27;100 | 0;0 |  |
|  | 31 | n | n | n | n | n | n | n | n | n | n | n | n | n | n | n | n | n | n | n | n | n | n | n | n | n | n | n | 0;0 | 27;100 | 0;0 |  |
|  | 32 | n | n | n | n | n | n | n | n | n | n | n | n | n | y | n | n | n | n | n | n | n | n | n | n | n | n | n | 1;4 | 26;96 | 0;0 |  |
|  | 33 | n | n | n | n | n | n | n | n | n | n | n | n | n | n | n | n | n | n | n | n | n | n | n | n | n | n | n | 0;0 | 27;100 | 0;0 |  |
| Dashboard function | 34 | n | n | n | n | n | n | n | n | n | n | n | n | n | n | n | n | n | n | n | n | n | n | n | n | n | n | n | 0;0 | 27;100 | 0;0 |  |
|  | 35 | n | n | n | n | n | n | n | n | n | n | n | n | n | n | n | n | n | n | n | n | n | n | n | n | n | n | n | 0;0 | 27;100 | 0;0 |  |
|  | 36 | n | n | n | n | n | n | n | n | n | n | n | n | n | n | n | n | n | n | n | n | n | n | n | n | n | n | n | 0;0 | 27;100 | 0;0 |  |
|  | 37 | n | n | n | n | n | n | n | n | n | n | n | n | n | y | n | n | n | n | n | n | n | n | n | n | n | n | n | 1;4 | 26;96 | 0;0 |  |
| Antimicrobial stewardship (AMS) | 38 | n | n | y | y | n | n | n | n | n | n | n | y | y | y | y | n | y | n | n | n | y | n | n | n | n | n | n | 8;30 | 19;70 | 0;0 |  |
|  | 39 | n | n | n | n | y | n | n | n | n | n | n | n | n | y | y | n | n | n | n | n | n | n | n | n | n | n | n | 3;11 | 24;89 | 0;0 |  |
|  | 40 | n | n | n | n | y | n | n | n | n | n | n | n | n | y | n | n | n | n | n | n | n | n | n | n | n | n | n | 2;7 | 25;93 | 0;0 |  |
|  | 41 | n | n | n | n | n | n | n | n | n | n | n | n | n | n | n | n | n | n | n | n | n | n | n | n | n | n | n | 0;0 | 27;100 | 0;0 |  |
|  | 42 | n | n | n | n | n | n | n | n | n | n | n | n | n | n | n | n | n | n | n | n | n | n | n | n | n | n | n | 0;0 | 27;100 | 0;0 |  |
|  | 43 | n | n | n | n | n | n | n | n | n | n | n | n | n | n | n | n | n | n | n | n | n | n | n | n | n | n | n | 0;0 | 27;100 | 0;0 |  |
| Notes and records | 44 | n | n | n | n | n | n | n | n | n | n | n | n | n | n | n | n | n | n | n | n | n | n | n | n | n | n | n | 0;0 | 27;100 | 0;0 |  |
|  | 45 | n | n | n | n | n | y | n | n | n | n | n | n | n | n | n | n | n | y | n | n | n | n | n | n | n | n | n | 2;7 | 25;93 | 0;0 |  |
|  | 46 | n | n | n | n | n | n | n | n | n | n | n | n | n | n | n | n | n | n | n | n | n | n | n | n | n | n | n | 0;0 | 27;100 | 0;0 |  |
|  | 47 | n | n | n | n | n | n | n | n | n | n | n | n | n | n | n | n | n | n | n | n | n | n | n | n | n | n | n | 0;0 | 27;100 | 0;0 |  |
|  | 48 | n | n | n | n | n | n | n | n | n | n | n | n | n | n | n | n | n | n | n | n | n | y | n | n | n | n | n | 1;4 | 26;96 | 0;0 |  |
|  | 49 | n | n | n | n | n | n | n | n | n | n | n | n | n | n | n | n | n | n | n | n | n | y | y | n | n | n | p | 2;7 | 24;89 | 1;4 |  |
| Network | 50 | n | n | n | n | n | n | n | n | n | n | n | n | n | n | n | n | n | n | n | n | n | n | n | n | n | n | n | 0;0 | 27;100 | 0;0 |  |
|  | 51 | n | n | n | n | n | n | n | n | n | n | n | n | y | y | y | n | n | n | n | y | n | n | n | n | n | n | n | 4;15 | 23;85 | 0;0 |  |
|  | 52 | n | n | n | n | n | n | n | n | n | n | n | n | n | n | n | n | n | n | n | n | n | n | n | n | n | n | n | 0;0 | 27;100 | 0;0 |  |
|  | 53 | n | n | n | n | n | n | n | n | n | n | n | n | n | n | n | n | n | n | n | n | n | n | n | n | n | n | n | 0;0 | 27;100 | 0;0 |  |
|  | 54 | n | n | n | n | n | n | n | n | n | n | n | n | n | n | n | n | n | n | n | n | n | n | n | n | n | n | n | 0;0 | 27;100 | 0;0 |  |
|  | 55 | n | n | n | n | n | n | n | n | n | n | n | n | n | n | n | n | n | n | n | n | n | n | n | n | n | n | n | 0;0 | 27;100 | 0;0 |  |
|  | 56 | n | n | n | n | n | n | n | n | n | n | n | n | n | n | n | n | n | n | n | n | n | n | n | n | n | n | n | 0;0 | 27;100 | 0;0 |  |
| Technical features | 57 | n | n | n | n | n | y | n | n | n | n | n | n | n | n | n | n | n | n | n | n | n | n | n | n | n | n | n | 1;4 | 26;96 | 0;0 |  |
|  | 58 | y | y | y | y | y | y | y | n | y | n | n | y | y | y | n | n | y | n | y | n | y | y | n | y | y | y | y | 19;70 | 8;30 | 0;0 |  |
|  | 59 | y | y | y | y | y | y | y | y | y | y | y | y | y | y | y | y | y | y | y | y | n | y | y | y | y | y | y | 26;96 | 1;4 | 0;0 |  |
|  | 60 | n | n | n | n | n | n | n | n | n | n | n | n | n | n | n | n | n | n | n | n | n | n | n | n | n | n | n | 0;0 | 27;100 | 0;0 |  |
|  | 61 | n | n | n | n | n | n | n | n | n | n | n | n | n | n | n | n | n | n | n | n | n | n | n | n | n | n | n | 0;0 | 27;100 | 0;0 |  |
|  | 62 | n | n | n | n | n | n | n | n | n | n | n | n | n | n | n | n | n | n | n | n | n | n | n | n | n | n | n | 0;0 | 27;100 | 0;0 |  |
|  | 63 | n | n | n | n | n | n | n | n | n | n | n | n | n | n | n | n | n | n | n | n | n | n | n | n | n | n | n | 0;0 | 27;100 | 0;0 |  |
|  | 64 | n | n | n | n | n | n | n | n | n | n | n | n | n | y | y | n | n | n | n | n | n | n | n | n | n | n | n | 2;7 | 25;93 | 0;0 |  |
|  | 65 | n | n | y | n | y | n | y | y | n | y | n | y | y | y | y | n | n | n | n | y | n | n | n | n | n | y | n | 11;41 | 16;59 | 0;0 |  |
|  | 66 | n | n | n | n | n | n | n | n | n | n | n | n | n | n | n | n | n | n | n | n | n | n | n | n | n | n | n | 0;0 | 27;100 | 0;0 |  |
|  | 67 | n | n | n | n | n | n | n | n | n | n | n | n | n | n | n | n | n | n | n | n | n | n | n | n | n | n | n | 0;0 | 27;100 | 0;0 |  |
|  | 68 | n | n | n | n | n | n | n | n | n | n | n | n | n | n | n | n | n | n | n | n | n | n | n | n | n | n | n | 0;0 | 27;100 | 0;0 |  |
|  | 69 | n | n | n | n | n | n | n | n | n | n | n | n | n | n | n | n | n | n | n | n | n | n | n | n | n | n | n | 0;0 | 27;100 | 0;0 |  |
|  | 70 | n | n | n | n | n | n | n | n | n | n | n | n | n | n | n | n | n | n | n | n | n | n | n | n | n | n | n | 0;0 | 27;100 | 0;0 |  |
|  | 71 | n | n | n | n | n | n | n | n | n | n | n | n | n | n | n | n | n | n | n | n | n | n | n | n | n | n | n | 0;0 | 27;100 | 0;0 |  |
|  | 72 | n | n | n | n | n | n | n | n | n | n | n | n | n | n | n | n | n | n | n | n | n | n | n | n | n | n | n | 0;0 | 27;100 | 0;0 |  |
|  | 73 | n | y | y | y | y | n | n | y | n | y | y | y | y | y | n | y | y | y | y | n | y | n | n | n | n | n | n | 15;56 | 12;44 | 0;0 |  |
|  | 74 | n | n | n | n | n | n | n | n | n | n | n | n | n | n | n | n | n | n | n | n | n | n | n | n | n | n | n | 0;0 | 27;100 | 0;0 |  |
|  | 75 | y | y | y | y | y | y | y | y | y | y | y | y | y | y | y | y | y | y | y | y | y | y | y | y | y | y | y | 27;100 | 0;0 | 0;0 |  |
|  | 76 | y | y | y | y | y | y | y | y | y | y | y | y | y | y | y | y | y | y | y | y | y | y | y | y | y | y | y | 27;100 | 0;0 | 0;0 |  |
|  | 77 | n | y | n | n | y | n | n | n | n | n | n | y | n | y | n | n | n | n | n | n | n | n | n | n | n | n | n | 4;15 | 23;85 | 0;0 |  |
|  | 78 | n | n | n | n | n | n | n | n | n | n | n | n | n | y | n | n | n | n | n | n | n | n | n | n | n | n | n | 1;4 | 26;96 | 0;0 |  |
|  | 79 | n | n | y | n | n | n | n | n | n | n | n | n | n | y | n | n | n | n | n | n | n | n | n | n | n | n | n | 2;7 | 25;93 | 0;0 |  |
|  | 80 | n | n | n | n | n | n | n | n | n | n | n | n | n | n | n | n | n | n | n | n | n | n | n | n | n | n | n | 0;0 | 27;100 | 0;0 |  |
|  | 81 | n | n | n | n | n | n | n | n | n | n | n | n | n | n | n | n | n | n | n | n | n | n | n | n | n | n | n | 0;0 | 27;100 | 0;0 |  |
|  | 82 | y | y | y | y | y | y | y | y | y | y | y | y | y | y | y | y | y | y | y | y | y | y | y | y | y | y | y | 27;100 | 0;0 | 0;0 |  |
|  | 83 | n | n | n | n | n | n | n | n | n | n | n | n | n | n | n | n | n | n | n | n | n | n | n | n | n | n | n | 0;0 | 27;100 | 0;0 |  |
|  | 84 | n | n | n | n | n | n | n | n | n | n | n | n | n | n | n | n | n | n | n | n | n | n | n | n | n | n | n | 0;0 | 27;100 | 0;0 |  |
|  | 85 | y | y | n | n | n | y | y | y | y | y | y | y | y | y | n | n | y | y | y | y | y | y | y | y | y | y | y | 22;81 | 5;19 | 0;0 |  |
|  | 86 | n | n | n | n | y | n | n | n | n | n | n | n | n | y | y | n | n | n | n | n | n | n | n | n | n | n | n | 3;11 | 24;89 | 0;0 |  |
| Tot y (n; %) | | 8;9 | 10;12 | 20;23 | 7;8 | 31;36 | 14;16 | 17;20 | 13;15 | 8;9 | 22;26 | 14;16 | 20;23 | 22;26 | 31;36 | 22;26 | 12;14 | 9;10 | 16;19 | 11;13 | 19;22 | 16;19 | 14;16 | 12;14 | 14;16 | 9;10 | 10;12 | 11;13 |  | | | |
| Tot n (n; %) | | 78;  9  1 | 76;88 | 66;77 | 79;92 | 55;64 | 72;84 | 69;80 | 73;85 | 78;91 | 64;74 | 72;84 | 66;77 | 64;74 | 54;63 | 63;73 | 74;86 | 77;90 | 70;81 | 75;87 | 67;78 | 70;81 | 72;84 | 74;86 | 72;84 | 77;90 | 76;88 | 72;84 |  |  |  |  |
| Tot p (n; %) | | 0;0 | 0;0 | 0;0 | 0;0 | 0;0 | 0;0 | 0;0 | 0;0 | 0;0 | 0;0 | 0;0 | 0;0 | 0;0 | 1;1 | 1;1 | 0;0 | 0;0 | 0;0 | 0;0 | 0;0 | 0;0 | 0;0 | 0;0 | 0;0 | 0;0 | 0;0 | 2;2 |  |  |  |  |

Appendix 1. Total number and percentage of accomplishments for each questionnaire item and globally for each of the 27 apps.
